# Tibial component with and without stem extension in a trabecular metal cone construct

**DOI:** 10.1007/s00167-016-4271-2

**Published:** 2016-09-03

**Authors:** Marrigje F. Meijer, Alexander L. Boerboom, Martin Stevens, Inge H. F. Reininga, Dennis W. Janssen, N. Verdonschot, Sjoerd K. Bulstra

**Affiliations:** 1 Department of Orthopaedics, University Medical Center Groningen, University of Groningen, PO Box 30.001, 9700 RB Groningen, The Netherlands; 2 Department of Trauma Surgery, University Medical Center Groningen, University of Groningen, Groningen, The Netherlands; 3 Orthopedic Research Laboratory, Radboud University Medical Center Nijmegen, Nijmegen, The Netherlands; 4 Laboratory of Biomechanical Engineering, University of Twente, Enschede, The Netherlands

**Keywords:** Total knee arthroplasty, Trabecular metal, Bone defect, Tibial component, TKA, TM, Stem extension, Cone

## Abstract

**Purpose:**

The purpose of this study was to investigate stability and strain distribution of a tibial plateau reconstruction with a trabecular metal cone while the tibial component is implanted with and without a stem, and whether prosthetic stability was influenced by bone mineral density. Trabecular metal cones are designed to fill up major bone defects in total knee arthroplasty. Tibial components can be implanted in combination with a stem, but it is unclear whether this is necessary after reconstruction with a trabecular metal cone. Implanting a stem can give extra stability, but may have negative side effects.

**Methods:**

Tibial revision arthroplasties with trabecular metal cones were performed after reconstruction of a 2B bone defect according to the Anderson Orthopedic Research Institute classification. Components were implanted in seven pairs of cadaveric tibiae; one tibia of each pair was implanted with stem and the other without. All specimens were loaded to one bodyweight alternating between the medial and lateral tibial component. Implant-bone micro-motions, bone strains, bone mineral density and correlations were measured and/or calculated.

**Results:**

Tibial components without a stem showed only more varus tilt [difference in median 0.14° (*P* < 0.05)], but this was not considered clinically relevant. Strain distribution did not differ. Bone mineral density only had an effect on the anterior/posterior tilt [*ρ*: −0.72 (*P* < 0.01)].

**Conclusion:**

Tibial components, with or without a stem, which are implanted after reconstruction of major bone defects using trabecular metal cones produce very similar biomechanical conditions in terms of stability and strain distribution. If in vivo studies confirm that a stem extension is not mandatory, orthopaedic surgeons can decide not to implant a stem.

**Level of evidence:**

II.

## Introduction

Major bone defects are frequently seen in revision total knee arthroplasty (rTKA). Reasons for this may be design and removal of the primary prosthesis, original disease process, mechanism of failure and technical problems during the procedure. Reconstruction of the knee joint and acquiring correct prosthetic alignment during rTKA therefore constitute a challenging task.

Types 2B and 3 bone defects according to the classification of the Anderson Orthopedic Research Institute (AORI) [13–15, 26] are commonly seen during rTKA and reconstruction of these major bone defects is usually done with metal augmentations in combination with a stem [6, 17, 27], which is shown to provide a mechanically stable reconstruction [10, 29, 33]. However, the literature shows that a stem may cause increased stress at the distal part of the stem and a decrease in stress at the proximal part [9]. If enhanced stress-shielding occurs, adverse bone remodelling may follow in the long term, possibly influencing component fixation and inducing fractures [5, 22, 36]. Another disadvantage of the use of stems is elevated stress at the tip of the stem, which is associated with pain, lower post-operative clinical outcome and increased risk of periprosthetic fracture [1].

Trabecular metal (TM) cones [7, 8] are a relatively new option for reconstruction of major bone defects during TKA. TM, made from tantalum, is reported to be biocompatible, corrosion-resistant and highly porous, with an average pore diameter of approximately 400 μm [2, 23]. Because of the porous structure ingrowth is encouraged when used uncemented, while fixation is solid when used with bone cement [4]. TM cones are available in various designs and sizes, in order to adjust to the type and size of the defect and bone. Several studies have shown good short-term functional results with evidence of osseointegration when a TM cone was used [11, 16, 20, 21, 25, 30, 35]. Tibial TM cones are designed to be impacted into the proximal tibia, to allow for osseous ingrowth and provide proximal support. After reconstruction of the proximal tibia, the tibial component is cemented in this cone and usually implanted with a stem. However, whether a stem is mandatory when a TM cone is used has not been investigated yet. A tibial TM cone is designed to enhance the carrying capacity of the metaphyseal bone of the proximal tibia, thereby rendering a situation as in a primary arthroplasty. Hence, a stem under the tibial component might not be needed to provide mechanical stability in combination with a tibial TM cone. Moreover, without use of a stem proximal stress-shielding may be reduced and it would save the costs of the stem.

The purposes of this study are thus to investigate stability and strain distribution of tibial reconstructions with a tibial component with and without a stem cemented on a TM cone. Additionally, it was investigated whether prosthetic stability was likely to be influenced by bone mineral density (BMD).

## Materials and methods

A cadaveric study was conducted in which seven pairs of fresh-frozen tibial bones [four males and three females, mean age 82 years (range 70–89)] were disarticulated at the ankle and stripped of all soft tissues. The distal ends of the tibiae were potted in bone cement. Bone mineral density (BMD) of the tibiae was determined using a calibrated CT scan and in-house software (DCMTK). Two spherical volumes of interest with a diameter of 11.4 mm in the medial and lateral proximal tibia were selected for the measurements. The averaged BMD of these two regions was later used to assess whether there was any effect of BMD on the biomechanical output parameters.

**Fig. 1:**
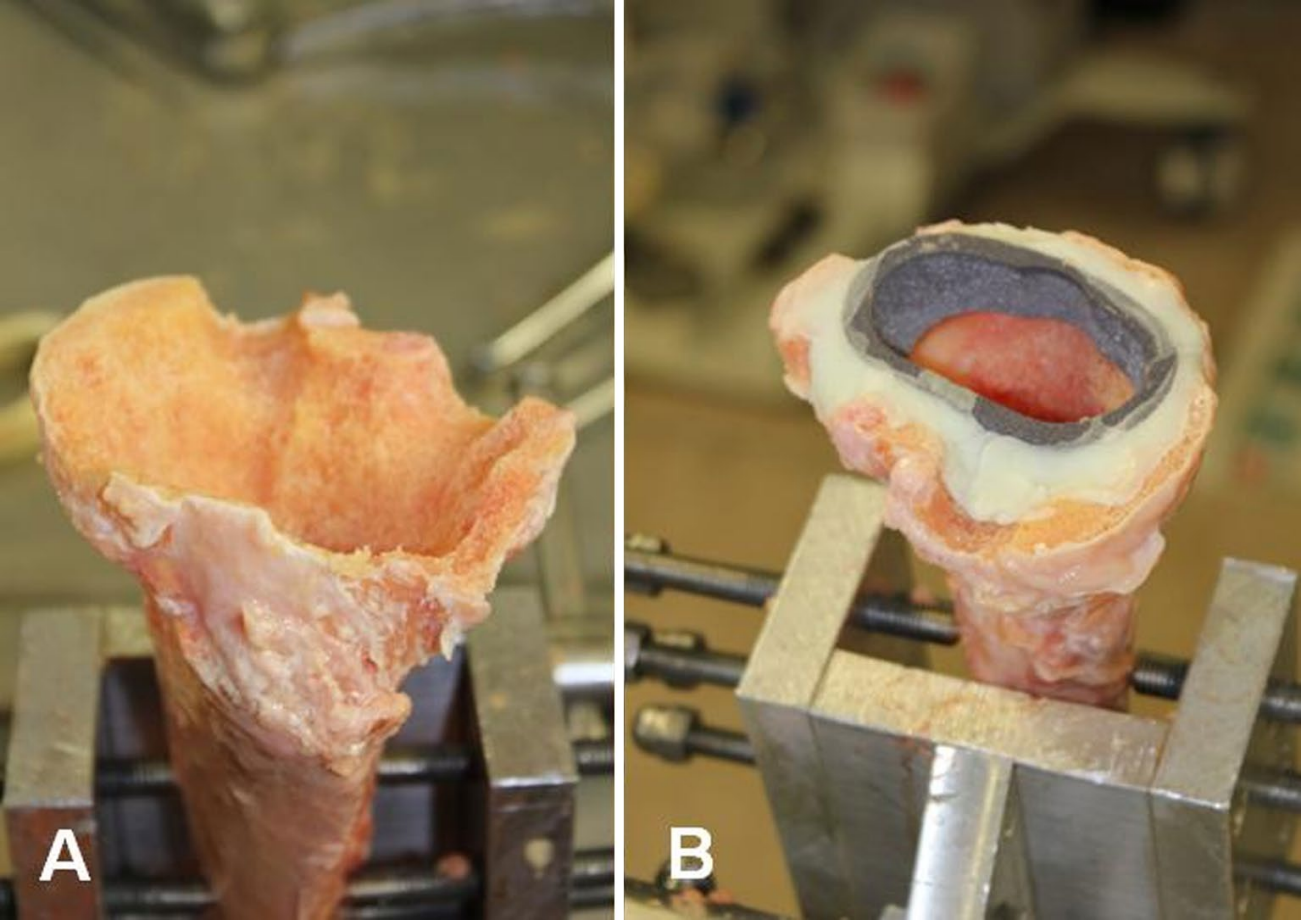
**a** An example of the created bone defect. **b** Reconstruction of a bone defect using a trabecular metal cone

In each tibia, bone cuts were made as if a primary knee prosthesis was to be placed. Secondly, an AORI type 2B defect [15] was created. On both the lateral and medial sides of the posterior rim of the tibia, a defect of approximately 1 cm^3^ was made. Furthermore, the proximal tibia was excochleated to simulate cancellous bone loss and the anteromedial rim was damaged, so finally a standard tibial component could not be fit stable on the cut surface, as demonstrated in Fig. 1a. In this way, the situation after removal of the primary prosthesis was simulated. The bone defect was first created in one cadaveric bone and served as a reference to create similar bone defects in the other cadaveric bones. For each pair of tibiae, one tibia was reconstructed using a porous tantalum metaphyseal full tibial cone [Trabecular Metal (TM), Zimmer Inc., Warsaw, IN, USA]. After this reconstruction a NexGen^®^ tibial component was implanted with a 100-mm press-fit stem extension (Zimmer Inc., Warsaw, IN, USA) and provided with a polyethylene insert [Legacy posterior stabilised (LPS) flex, 10–12 mm; Zimmer Inc., Warsaw, IN, USA]. The other tibia was reconstructed using the same TM cone and NexGen^®^ tibial component and polyethylene insert, but no stem extension was used. All tibial components were cemented using bone cement (Refobacin^®^ revision bone cement with clindamycin and gentamicin, Biomet Inc., Warsaw, IN, USA). Only the proximal part of the tibial component up to the connection of the stem was cemented. As bone ingrowth is impossible in this model and TM cones are frequently implanted using bone cement in clinical practice, the TM cones were also fixed by using bone cement in our experimental set-up (Fig. 1). Preparation of the tibiae and implantation of the components was performed by one orthopaedic surgeon (ALB). Allocation of whether the left or the right tibia was implanted with a stem was randomised by using a computer-generated list.

**Fig. 2:**
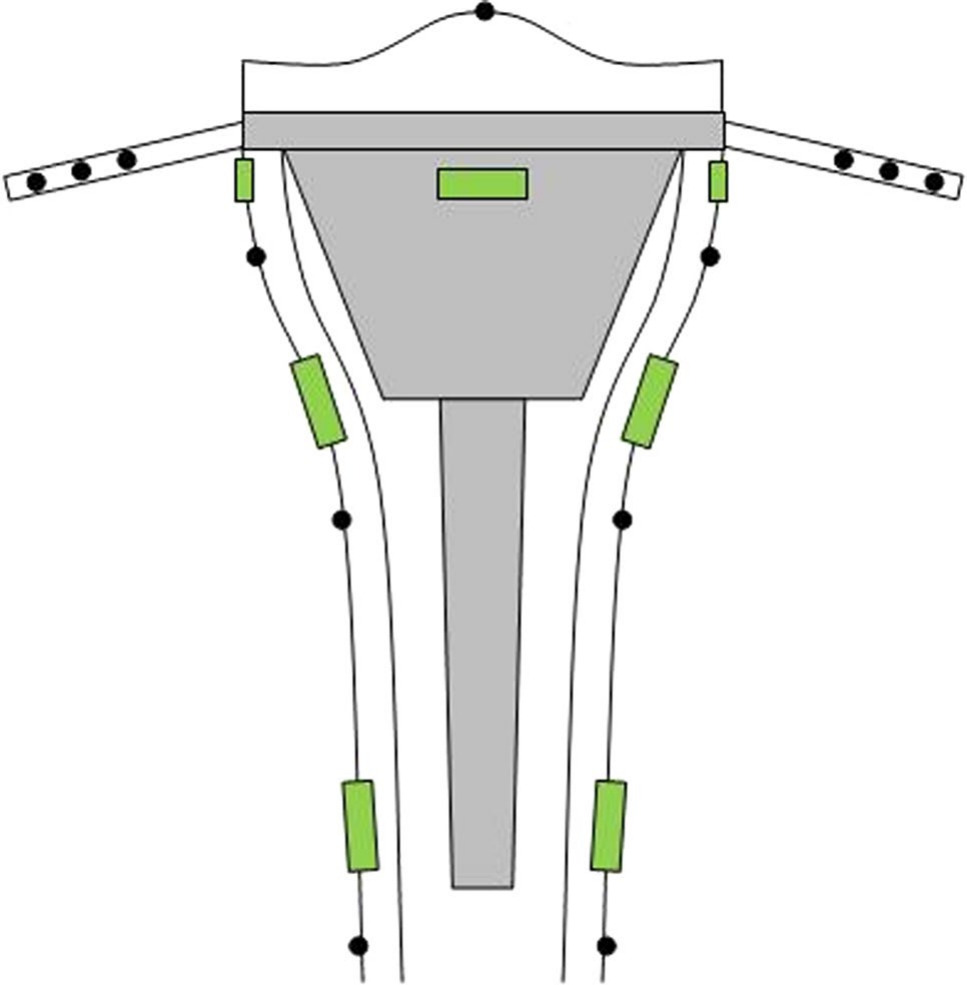
Schematic representation of the locations of the tantalum pellets and strain gauges. The *black dots* represent the tantalum pellets and the *green rectangles* represent the strain gauges

The next step was to test the cadaveric reconstructions for stability. RSA was used to determine migration and rotation of the components. Seven tantalum pellets (0.8 mm diameter) were glued to the tibial component and six tantalum pellets were placed into the shaft of the proximal and distal tibia in standard positions (Fig. 2). The tip of the polyethylene insert was chosen as the origin of the coordinate system relative to which rotations and translations of the component were expressed. Stereoradiograms of the medially and laterally loaded situations were made before loading and after 10,000 loading cycles. The radiograms were digitised manually and analysed using RSA software (RSA-CMS, Medis, Leiden, The Netherlands). In a previously conducted knee study, the estimated error for the same RSA analysis was less than 50 µm for repeated measurements, with a standard deviation of 0.1 mm [3]. Endpoints of the RSA were translation and rotation along the *X*-, *Y*- and *Z*-axes. Translations along these axes were defined as medial/lateral translation, superior/inferior translation and anterior/posterior translation, respectively. Rotations along the *X*-, *Y*- and *Z*-axes were defined as anterior/posterior tilt, internal/external rotation and varus/valgus tilt, respectively. Total translation (TT) was also calculated using the following equation: TT = √(*x* ^2^ + *y* ^2^ + *z* ^2^) [32]. The TT can be considered as a close equivalent to maximum total point motion (MTPM) [32, 34]. We calculated the TT because the MTPM was not calculated with the RSA software used in this study.

To evaluate strain distributions between the tibiae with and without stem, seven strain gauges (type YFLA-5, Tokyo Sokki Kenkyujo Co., Ltd., Tokyo) were used. The gauges were positioned horizontally on the cortex 15 mm under the tibial component at the medial, anterior and lateral side, and vertically at the connection of the TM cone to the stem and 5 mm proximally of the tip of the stem on the medial and lateral sides (Fig. 2). The contralateral tibia served as reference for the tibiae in which no stem was used. All strain gauges were connected to an amplifier and a computer to record data using monitoring software (quickDAQ 1.5.0.6, Data Translation, Inc, Marlboro, MA, USA). Strain was recorded during the entire loading session of 10,000 cycles.

**Fig. 3:**
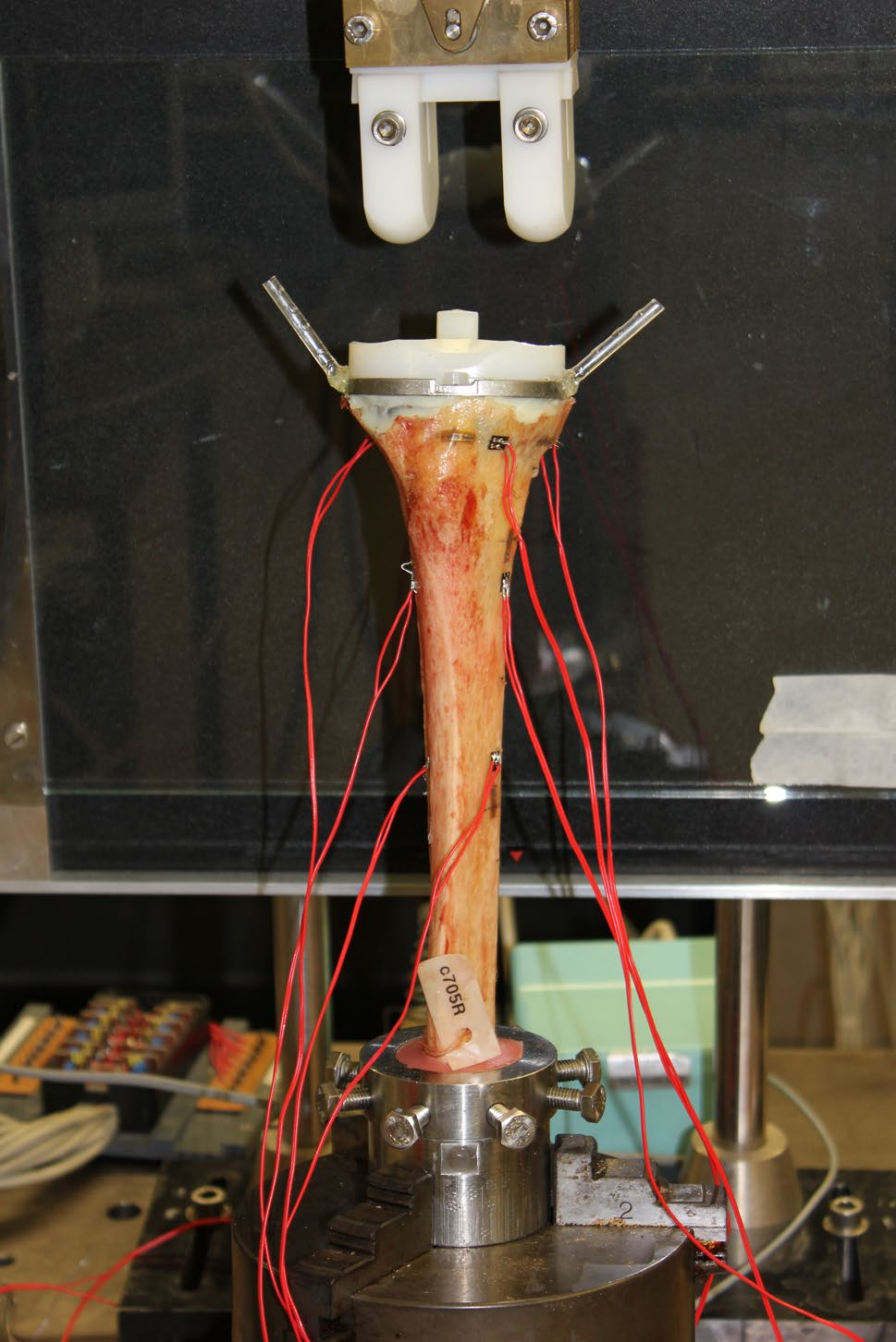
Experimental set-up

The tibiae were clamped into a testing machine (MTS, model 458020, MTS Systems Corporation, Minneapolis, MN, USA) with the tibial plateau parallel to the working bench (Fig. 3). A unicondylar axial load alternating between the medial and lateral parts of the tibial plateau was performed. The load cycled between zero and 700 N at a frequency of 1 Hz in a series of eight loading cycles, i.e. the medial and lateral parts of the tibial plateau were both loaded eight times, thus applying varus–valgus stress to test for maximal instability of the reconstruction. A previous study at the same institution was conducted with the same set-up [24].

In accordance with regulations of the Medical Ethical Review Board of University Medical Center Groningen, ethical approval for this study was not indicated since the study was performed using cadaveric bones.

### Statistical analyses

All statistical analyses were performed using the PASW software package (version 19, SPSS, Chicago). Potential differences between the two groups in rotation, translation and TT for both the medially and laterally loaded situations after 10,000 cycles were investigated using the paired Wilcoxon signed-rank test. The absolute differences between the medially and laterally loaded RSA measurements and the TT for these differences were also compared between the two groups using the paired Wilcoxon signed-rank test. We hypothesised that the difference in rotation or translation between the medially and laterally loaded stereoradiograms may serve as a measure of instability of the construction. The paired Wilcoxon signed-rank test was also used to compare the minimal and maximal strains at the different levels between the tibiae with and without stem. The difference between the minimal and maximal strains per level in the last 32 cycles (equal to four alternating configurations of medial and lateral load) of the loading session was also compared. Spearman’s correlation coefficient (*ρ*) was used to determine the correlation between bone mineral density and the differences in rotation, translation and TT between the medially and laterally loaded situations. The correlation coefficients were interpreted according to the benchmarks described by Domholdt [12]: *ρ* 0.90–1.00 represents a very strong correlation, 0.70–0.89 a strong correlation, 0.50–0.69 moderate, 0.26–0.49 weak and 0.00–0.25 little if any correlation [12]. A *P* value <0.05 was considered to indicate statistical significance.

## Results

Implanting the prosthetic material caused a fissure in one of the proximal tibiae. During loading, the proximal part of this tibia broke off. This tibia and the matching contralateral tibia were therefore excluded from further analysis. None of the other tibial components migrated visually during the loading sessions; hence, data of six pairs of tibiae were used for further analysis.

No significant differences were found for anterior/posterior tilt or internal/external rotation between both groups after 10,000 cycles (Table 1). For varus/valgus tilt, a minimal yet significant difference was found for the laterally loaded RSA measurements. The group without a stem showed more varus tilt than the group with a stem after 10,000 cycles (*P* = 0.046). No significant difference was found for the medially loaded RSA measurements (Table 1). Translation in the three directions and TT showed no significant differences between the two groups (Table 1). When comparing the medially and laterally loaded stereoradiograms after 10,000 cycles, no significant differences in rotation or translation in any of the three directions were found (Table 2). TT did not show any significant differences between the two groups when comparing the medially and laterally loaded RSA measurements (Table 2).

**Table 1:** Median, minimal and maximal rotations and translations in the three axes and total translation (TT) after 10,000 cycles

Anterior/posterior tilt						Internal/external rotation				Varus/valgus tilt			
Stem/no stem	Loading	Median	Min	Max	*P* value	Median	Min	Max	*P* value	Median	Min	Max	*P* value
Stem	Medially	0.08	0.04	0.53		−0.01	−0.42	0.04		−0.07	−0.19	0.11	
No stem	Medially	−0.06	−1.38	0.33	n.s.	0.05	−0.16	0.18	n.s.	0.06	−0.36	0.74	n.s.
Stem	Laterally	0.06	−0.11	0.24		0.02	−0.26	0.09		−0.05	−0.16	0.04	
No stem	Laterally	−0.00	−1.39	0.34	n.s.	0.09	−0.17	0.39	n.s.	0.09	−0.09	0.79	<0.05*

Medial/lateral translation						Superior/inferior translation				Anterior/posterior translation				TT			
Stem/no stem	Loading	Median	Min	Max	*P* value	Median	Min	Max	*P* value	Median	Min	Max	*P* value	Median	Min	Max	*P* value
Stem	Medially	0.07	−0.06	0.23		0.19	−0.18	0.49		0.14	0.43	−0.13		0.35	0.13	0.62	
No stem	Medially	−0.01	−0.24	0.38	n.s.	−0.00	−0.32	0.97	n.s.	0.05	0.26	−0.17	n.s.	0.36	0.06	0.98	n.s.
Stem	Laterally	0.01	−0.17	0.16		0.06	−0.32	0.21		0.03	0.31	−0.19		0.34	0.01	0.43	
No stem	Laterally	−0.19	−0.29	0.23	n.s.	0.04	−0.26	0.13	n.s.	0.01	0.34	−0.27	n.s.	0.29	0.19	0.71	n.s.

Rotation is stated in degrees (°) and translation in mm

*n.s.* not significant

* Significant difference

**Table 2:** Median, minimal and maximal differences between the medially and laterally loaded RSA measurements after 10,000 cycles for rotations and translations in the three axes and total translation (TT)

	Stem/no stem	Median	Minimum	Maximum	*P* value
Anterior/posterior tilt	Stem	0.11	0.01	0.29	
Anterior/posterior tilt	No stem	0.01	0.00	0.58	n.s.
Internal/external rotation	Stem	0.06	0.01	0.18	
Internal/external rotation	No stem	0.20	0.09	0.34	n.s.
Varus/valgus tilt	Stem	0.10	0.00	0.23	
Varus/valgus tilt	No stem	0.13	0.05	0.38	n.s.
Medial/lateral translation	Stem	0.12	0.04	0.33	
Medial/lateral translation	No stem	0.17	0.05	0.61	n.s.
Superior/inferior translation	Stem	0.25	0.01	0.58	
Superior/inferior translation	No stem	0.20	0.02	1.24	n.s.
Anterior/posterior translation	Stem	0.09	0.01	0.45	
Anterior/posterior translation	No stem	0.09	0.00	0.15	n.s.
TT	Stem	0.29	0.19	0.71	
TT	No stem	0.38	0.13	1.25	n.s.

Rotation is stated in degrees (°) and translation in mm

*n.s.* not significant

A similar strain pattern was found for all strain gauges. Neither minimal strains (dip of line chart) nor maximal strains (peak of line chart) showed any significant difference between both groups for any of the locations (proximally, connection of cone to the stem or distally) (Table 3). No significant differences were found regarding the difference between the minimal and maximal strains (differences between peak and dip of line chart) between the two groups (Table 4).

**Table 3:** Median, minimum and maximum of the minimal and maximal strains measured during the last 32 cycles of the loading session

Stem/no stem	Cycles	Strain	Median	Minimum	Maximum	*P* value
*Strains proximally (medial, anterior and lateral)*						
Stem	10,000	Medial min	39.57	−97.62	145.11	
No stem	10,000	Medial min	27.70	−153.02	332.42	n.s.
Stem	10,000	Medial max	76.51	−21.11	179.40	
No stem	10,000	Medial max	39.57	−306.04	129.28	n.s.
Stem	10,000	Anterior min	19.79	−142.47	203.15	
No stem	10,000	Anterior min	25.06	−108.17	253.28	n.s.
Stem	10,000	Anterior max	−42.21	−108.17	60.68	
No stem	10,000	Anterior max	26.38	−163.57	150.38	n.s.
Stem	10,000	Lateral min	146.42	−5.28	1155.57	
No stem	10,000	Lateral min	26.38	−1216.25	1290.12	n.s.
Stem	10,000	Lateral max	178.08	65.96	736.08	
No stem	10,000	Lateral max	40.89	−817.87	284.93	n.s.
*Strains at the level of the connection of cone to stem (medial and lateral*)						
Stem	10,000	Medial min	48.81	−282.30	356.17	
No stem	10,000	Medial min	110.81	−36.94	530.29	n.s.
Stem	10,000	Medial max	87.06	60.68	332.42	
No stem	10,000	Medial max	75.19	−18.47	1408.84	n.s.
Stem	10,000	Lateral min	−7.92	−124.00	517.10	
No stem	10,000	Lateral min	7.92	−226.89	110.81	n.s.
Stem	10,000	Lateral max	34.30	−81.79	229.53	
No stem	10,000	Lateral max	17.15	−197.87	514.46	n.s.
*Strains distally (medial and lateral)*						
Stem	10,000	Medial min	0.00	−44.85	274.38	
No stem	10,000	Medial min	−1.32	−522.38	44.85	n.s.
Stem	10,000	Medial max	97.62	34.30	498.63	
No stem	10,000	Medial max	75.19	−2.64	321.87	n.s.
Stem	10,000	Lateral min	−48.81	−279.66	31.66	
No stem	10,000	Lateral min	−30.34	−139.83	0.00	n.s.
Stem	10,000	Lateral max	27.70	−29.02	63.32	
No stem	10,000	Lateral max	19.79	−34.30	474.89	n.s.

Strain is stated in micro-strains (µstrain)

*n.s.* not significant

**Table 4:** Median, minimal and maximal difference in strain between the minimal and maximal strains measured during the last 32 cycles of the loading session

Location of strain gauge	Stem/no stem	Median	Minimum	Maximum	*P* value
Proximal medial	Stem	−13.19	−176.77	42.21	
Proximal medial	No stem	64.64	−23.75	203.15	n.s.
Proximal anterior	Stem	22.43	−68.60	142.47	
Proximal anterior	No stem	50.13	−47.49	102.89	n.s.
Proximal lateral	Stem	5.28	−271.74	419.49	
Proximal lateral	No stem	−14.51	−398.38	1005.18	n.s.
Cone-stem medial	Stem	−30.34	−377.27	52.77	
Cone-stem medial	No stem	−9.23	−878.55	68.60	n.s.
Cone-stem lateral	Stem	−21.11	−182.04	287.57	
Cone-stem lateral	No stem	−27.70	−461.70	2.64	n.s.
Distal medial	Stem	−39.57	−532.93	168.85	
Distal medial	No stem	−52.77	−844.25	13.19	n.s.
Distal lateral	Stem	−58.04	−342.98	0.00	
Distal lateral	No stem	−50.13	−614.72	2.64	n.s.

Strain is stated in micro-strains (µstrain)

*n.s.* not significant

Mean BMD of the cadavers was 115 mg/mm^3^ (SD 64; range 30–213). The tibia that fractured during loading had a BMD of 113 mg/mm^3^. As mentioned earlier, this tibia and the matching contralateral tibia were excluded from further analysis. The correlation between the BMD and the difference between the medially and laterally loaded RSA measurements for anterior/posterior tilt was strong (*ρ*: −0.72, *P* < 0.01), indicating that for this direction more motion was produced for lower-density bones. After exclusion of one outlier, correlation was moderate and significant (*ρ*: −0.64, *P* < 0.04), and little if any correlation existed for internal/external rotation and varus/valgus tilt [*ρ*: <−0.10, *P* = (n.s.)]. There was little if any correlation between the BMD and the difference between the medially and laterally loaded RSA measurements for medial/lateral translation [*ρ*: −0.21, *P* = (n.s.)], a moderate and non-significant correlation for superior/inferior translation [*ρ*: −0.50, *P* = (n.s.)], and a weak and non-significant correlation for anterior/posterior translation [*ρ*: −0.25, *P* = (n.s.)]. The correlation between the BMD and the TT of the difference between the medially and laterally loaded measurements was moderate and non-significant [*ρ*: −0.50, *P* = (n.s.)].

## Discussion

The most important finding of the present study was that tibial components, with or without a stem, which are implanted after reconstruction of major bone defects using TM cones produce very similar biomechanical conditions in terms of stability and strain distribution. TM cones are designed to fill up bone defects during TKA. However, it is unclear whether tibial components should be implanted with or without a stem after reconstruction of bone defects (AORI 2B/3) using a TM cone. The aim was therefore to investigate stability and strain distribution of a tibial plateau reconstruction with a TM cone while the tibial component was implanted with and without a stem. We also questioned whether prosthetic stability was influenced by BMD.

Results of this study showed no evidence that a stem creates benefit and improves stability when a tibial component is implanted in a TM cone. Results of RSA measurements showed no difference between both groups, indicating that both constructions are stable. To our knowledge, this is the first study to investigate stability of a tibial reconstruction with a TM cone and a tibial component without stem extension. Results of RSA analysis only showed a significant difference for varus/valgus tilt of the laterally loaded RSA measurements. The cone without a stem showed more varus tilt than the cone implanted with a stem. Differences were small though. The difference between the medians of both groups was only 0.14°, and the range was 0.20° and 0.89° for tibial components with and without a stem, respectively. Since these differences were extremely small, they obviously lacked clinical significance. Rotations and translations in the other directions did not show significant differences between the two groups. We calculated differences between the medially and laterally loaded RSA measurements, as this may also give an indication of instability, but this showed no differences either.

Advantages of using stems are resistance of the tibial component to shear loads, reduced tibial lift-off and increased stability leading to reducing micro-motion. Potential disadvantages include stress-shielding with associated reduction in BMD, risk of subsidence and loosening, periprosthetic fracture and end-of-stem pain [31]. In vitro studies have demonstrated a decrease in proximal tibial strain and increase in strain at the distal tip of the stem when a stem was used [5, 9]. In a study by Bourne et al. [5], it was concluded that tibial components should have either no short intramedullary stem or only a short one, due to negative side effects. In our study, we did not find differences in strain distribution between the two groups. Reason for this may be that we analysed bones with severe defects and reconstructed those with the cones. Apparently the addition of the stem did not add to stability or to load transfer; therefore, no strain increase at the tip of the stem or decrease at the proximal tibia was observed. This finding is consistent with our results of the RSA analysis, showing that a base plate without stem in a TM cone is a stable mechanical construction.

In this study, a low BMD strongly correlated with a larger difference between the medially and laterally loaded RSA measurements for anterior/posterior tilt. We hypothesised that a greater difference between these measurements could be an indication of instability. A low BMD may thus theoretically decrease stability of the construction. Even though the correlation between the BMD and anterior/posterior tilt was strong and significant, maximum difference in anterior/posterior tilt was only 0.58°. Such differences are very small and not considered clinically important. For other rotations and translations, correlations with BMD ranged from moderate to weak and were non-significant. It is therefore assumed that the results of this study are representative. Patients who undergo rTKA in general practice tend to be older, so variety in BMD can also be expected. Moreover, analyses were done in pairs (left and right tibia) per cadaver, thereby facilitating investigation of the effect of the stem despite the variability in BMD.

This study has some limitations. First of all, design was a biomechanical in vitro study using cadaveric bone. Forces and number of cycles applied are a simplification of the situation in vivo. Notwithstanding, the aim of this study was to investigate stability of the proximal tibia after bone defect repair with a tibial TM cone, for which this set-up is a suitable design. Several in vivo studies have reported good short-term functional and radiological outcome after reconstruction of bone defects using TM cones in TKA with use of stem extensions [11, 18, 19, 28, 35]. And yet, in vivo studies have to be conducted to gain insight into radiological and functional effects when a tibial component is implanted without a stem after reconstruction with a TM cone. Secondly, tibial TM cones in this study were implanted using bone cement. The porous structure of TM encourages bone ingrowth and can be implanted without the use of cement. From clinical experience, we have found that around 50 % of the implanted tibial TM cones in our hospital use bone cement. An uncemented cone has to fit exactly when implanted; otherwise, cement has to be used. Since bone ingrowth could obviously not happen in this study and TM cones are also placed using bone cement in clinical practice, we decided to implant all TM cones using bone cement. In this way, homogeneity of the procedure is achieved and one could imagine interpreting the findings for the cementless TM cone applications as if ingrowth had occurred—as would be the case in clinical practice. Thirdly, the cadaveric bones used varied in BMD, and age of the cadavers was relatively old. This is inherent to the use of cadaveric bone, but also similar to the patient population of rTKA. BMD appeared to influence only anterior/posterior tilt. Stability in other directions was not influenced.

## Conclusion

This study suggests that additional stem extension of the tibial component may not be required. *In vivo* studies have to be performed to gain insight into the radiological and functional effects when a tibial component is implanted without a stem after reconstruction with a TM cone. If in vivo studies confirm that a stem extension is not mandatory, orthopaedic surgeons can decide not to implant a stem.
